# Kikuchi-Fujimoto disease with hemophagocytic lymphohistiocytosis in children: a seven-patient case series

**DOI:** 10.3389/fimmu.2026.1935403

**Published:** 2026-09-30

**Authors:** Wen-ling Guo, Dan-ping Zhu, Li Huang, Qiu-yan Peng, Pei-qing Li, Song Zhang, De-feng Liang

**Affiliations:** 1 Department of Nutrition, Guangzhou Women and Children’s Medical Center, Guangzhou Medical University, Guangdong Provincial Clinical Research Center for Child Health, Guangzhou, Guangdong, China; 2 Department of Emergency, Guangzhou Women and Children’s Medical Center, Guangzhou Medical University, Guangdong Provincial Clinical Research Center for Child Health, Guangzhou, Guangdong, China; 3 Department of Allergy, Immunology and Rheumatology, Guangzhou Women and Children’s Medical Center, Guangzhou Medical University, Guangdong Provincial Clinical Research Center for Child Health, Guangzhou, China

**Keywords:** child, hemophagocytic lymphohistiocytosis, histiocytic necrotizing lymphadenitis, Kikuchi-Fujimoto disease, systemic lupus erythematosus

## Abstract

**Background:**

Kikuchi-Fujimoto disease, also known as histiocytic necrotizing lymphadenitis (HNL), is typically self-limited but may be complicated by hemophagocytic lymphohistiocytosis (HLH) and may coexist with autoimmune disease, particularly systemic lupus erythematosus (SLE). Pediatric HNL-HLH is rare, and detailed data on pathology, HLH-2004 criteria, immune profiles, acute-care requirements, and subsequent autoimmune diagnoses remain limited.

**Methods:**

We retrospectively reviewed children with biopsy-confirmed HNL who fulfilled at least five of eight HLH-2004 criteria at Guangzhou Women and Children’s Medical Center from January 2020 to December 2025. We summarized patient-level clinical, laboratory, immunological, pathological, treatment, acute-outcome, and follow-up data.

**Results:**

Seven children were included (four boys; median age, 7 years; range, 1–13 years). Three patients met seven HLH-2004 criteria, two met six, and two met five. Fever, ≥2-lineage cytopenia, hyperferritinemia, and hypertriglyceridemia and/or hypofibrinogenemia occurred in all patients. Antinuclear antibody was positive in three patients, and complement C3 and/or C4 levels were low in four. Admission peripheral-blood natural killer (NK)-cell proportions were below the local reference range in five patients (range, 1.11%–9.83%). Four patients required pediatric intensive care, and three underwent plasma exchange. At discharge, complete remission was documented in six patients and partial remission in one; no in-hospital deaths occurred. Six patients had post-discharge follow-up (median, 23.5 months; range, 7–73 months), with no documented HLH recurrence. SLE was diagnosed in three patients during the index hospitalization, at 6, 11, and approximately 92 days after admission.

**Conclusion:**

This seven-patient series documents intensive-care requirements in four children with HNL-HLH and clinical SLE diagnoses in three during the index hospitalization. These observations highlight the importance of pathological confirmation, assessment of hyperinflammatory severity, and rheumatologic follow-up when autoimmune features are present. Larger multicenter studies are needed to clarify the prognostic and therapeutic implications of immune-profile abnormalities.

## 1 Introduction

Kikuchi-Fujimoto disease, also known as histiocytic necrotizing lymphadenitis (HNL), is an uncommon necrotizing lymphadenopathy with an incompletely defined etiology. It classically presents with fever and tender cervical lymphadenopathy in young adults of Asian ancestry, but cases occur worldwide and in children (1–3). Diagnosis rests on lymph node histology, including paracortical expansion, karyorrhectic necrosis, and a CD68/CD163-positive histiocytic infiltrate in the absence of neutrophil-rich suppuration. Although HNL is usually self-limited, severe systemic presentations occur. Immunopathological studies have implicated plasmacytoid dendritic cells and cytotoxic T cells, and HNL may coexist with or precede systemic autoimmune disease, particularly systemic lupus erythematosus (SLE) (4–7).

Hemophagocytic lymphohistiocytosis (HLH) is a clinically defined hyperinflammatory syndrome characterized by sustained T-cell and macrophage activation. In the absence of a molecular diagnosis, the HLH-2004 framework defines HLH by fulfillment of at least five of eight criteria (8). These comprise fever, splenomegaly, ≥2-lineage cytopenia, hypertriglyceridemia and/or hypofibrinogenemia, hemophagocytosis in bone marrow, spleen, or lymph nodes, low or absent natural killer (NK)-cell activity, hyperferritinemia, and elevated soluble CD25 (sCD25). Experimental studies implicate CD8-positive T cells and IFN-γ-driven macrophage activation in HLH. Pediatric secondary HLH may also show prominent IFN-γ and IL-10 elevations compared with the broader cytokine patterns observed in sepsis (9–11).

The overlap between HNL and HLH (HNL-HLH) is uncommon, and pediatric evidence remains limited to case reports, literature reviews, and small retrospective series. A retrospective series of 13 children from a tertiary hospital in China reported heterogeneous clinical presentations and autoimmune disease in a minority of survivors during follow-up (12). Macrophage activation syndrome has also been reported as a complication of pediatric HNL in a separate cohort (13). In an adult five-patient HNL-HLH series, two patients were diagnosed with SLE during follow-up (14). Together, these reports suggest clinical overlap among necrotizing lymphadenitis, hyperinflammation, and autoimmunity, but the frequency and timing of autoimmune manifestations in children remain uncertain.

Viral triggers represent another unresolved issue. Epstein-Barr virus (EBV) has been proposed in HNL and in some HNL-HLH reports, yet meta-analytic and tissue-based studies have not established a consistent EBV or herpesvirus association (15–18). Recent lymph-node RNA-sequencing and interferon studies have linked HNL to aberrant type I interferon activity (19, 20). This may provide an immunological context for reported HNL-SLE associations, although no single causal pathway has been established. Detailed pediatric case-level reporting across virology, cytokines, lymphocyte subsets, treatment intensity, and SLE timing is therefore still needed.

To address these gaps, we retrospectively analyzed seven children with biopsy-confirmed HNL-HLH managed at a single tertiary pediatric center over six years. We characterized patient-level HLH-2004 fulfillment, EBV and cytomegalovirus testing, cytokine and lymphocyte-subset profiles, treatment and organ support, acute outcomes, and concurrent or subsequent SLE diagnoses.

## 2 Materials and methods

### 2.1 Study design and data collection

Between January 2020 and December 2025, 78 patients aged <18 years were diagnosed with HNL at Guangzhou Women and Children’s Medical Center, Guangzhou Medical University. All 78 patients were screened for HLH, and seven fulfilled the HLH-2004 diagnostic criteria and were included in this single-center retrospective case series. HNL was confirmed by lymph node biopsy in all seven included patients. Medical records were reviewed for demographic characteristics, clinical manifestations, laboratory and immunological findings, lymph node pathology, virological testing, and treatment. Pediatric intensive care unit (PICU) admission, discharge outcome, follow-up duration, HLH recurrence, and SLE outcome were also recorded. This retrospective study was approved by the Ethics Review Committee of Guangzhou Women and Children’s Medical Center, Guangzhou Medical University (Approval No. 225A01; April 27, 2026). Written informed consent permitting the use of de-identified clinical data for research and publication was obtained from the parents or legal guardians of all participants.

Laboratory variables were recorded as peak or nadir values during the index hospitalization, as clinically appropriate. Cytokine and lymphocyte-subset results were obtained at the index admission and interpreted using institutional reference ranges. Missing variables were reported descriptively, and no data were imputed.

### 2.2 Diagnostic criteria

HNL was diagnosed on lymph node biopsy based on paracortical expansion, patchy karyorrhectic necrosis, abundant nuclear debris, histiocytic infiltration, and absence of neutrophil-rich suppuration. Immunohistochemistry, when available, was used to support the diagnosis and exclude lymphoma.

HLH was defined by fulfillment of at least five of the eight HLH-2004 criteria: fever, splenomegaly, cytopenia affecting at least two lineages, hypertriglyceridemia and/or hypofibrinogenemia, hemophagocytosis, low or absent NK-cell activity, ferritin ≥500 μg/L, and soluble CD25 (sCD25) ≥2,400 U/mL (8).

Clinical SLE diagnoses were based on pediatric rheumatology assessments documented in the medical records. Available autoimmune serology, complement levels, and relevant clinical features were summarized descriptively.

### 2.3 Statistical analysis

Continuous variables were summarized as median (range), and categorical variables as number/total. Given the small sample size, all analyses were descriptive, and no inferential statistical tests were performed.

## 3 Results

### 3.1 Demographic and clinical characteristics

Demographic and clinical characteristics are shown in **Table 1**. The cohort comprised four male and three female patients. The median age was 7 years (range, 1–13); one patient (Case 3) was aged 1 year. Median hospitalization duration was 30 days (range, 29–103). All patients had fever, with a median peak temperature of 40.0°C (range, 39.8–41.0°C). Lymphadenopathy was present in all patients, with a median largest lymph node diameter of 2.0 cm (range, 1.5–3.5 cm); cervical and inguinal nodes were most frequently involved. In all seven patients, lymphadenopathy preceded the diagnosis of HLH. The intervals from the onset of lymphadenopathy to HLH diagnosis were 67, 12, 17, and 15 days in Cases 1, 5, 6, and 7, respectively; the exact intervals could not be determined in Cases 2–4. Rash occurred in 4 of 7 patients, hepatomegaly in 6 of 7, and splenomegaly in 5 of 7. Respiratory tract bleeding occurred in Case 1 and gastrointestinal bleeding in Case 4; central nervous system involvement was recorded in Case 4.

**Table 1:** Clinical characteristics, laboratory findings, and outcomes of the cohort.

Variable	Value
Number of patients	7
Male sex	4/7
Age, years	7 (1–13)
Hospital stay, days	30 (29–103)
Peak temperature, °C	40.0 (39.8–41.0)
Lymphadenopathy	7/7
Largest lymph node diameter, cm	2.0 (1.5–3.5)
Cervical lymphadenopathy	5/7
Inguinal lymphadenopathy	4/7
Rash	4/7
Hepatomegaly	6/7
Splenomegaly	5/7
CNS involvement	1/7
Bleeding manifestations	2/7
Hemoglobin nadir, g/L	64 (52–69)
Platelet nadir, ×10^9^/L	51 (20–120)
Absolute neutrophil count nadir, ×10^9^/L	0.64 (0.05–6.06)
Ferritin peak, ng/mL	8,205 (1,228–16,500)
Triglyceride peak, mmol/L	5.11 (2.29–5.68)
Fibrinogen nadir, g/L	1.22 (0.84–2.48)
ALT peak, U/L	361 (95–2,525)
AST peak, U/L	478 (120–7,960)
LDH peak, U/L	1,851 (573–2,809)
Albumin nadir, g/L	26.3 (19.9–28.8)
sCD25, U/mL	17,744 (3,474–43,308), n=5
PICU admission	4/7
Plasma exchange	3/7
Mechanical ventilation	2/7
Vasoactive drug support	1/7
Complete remission at discharge	6/7
In-hospital death	0/7
Post-discharge follow-up available	6/7
Follow-up duration, months	23.5 (7–73), n=6
Documented HLH recurrence during follow-up	0/6
Clinical SLE diagnosis	3/7

Data are presented as n/N or median (range). Laboratory values indicate peak or nadir values during the index hospitalization, as clinically appropriate. Acute remission status was recorded according to the treating team’s assessment at discharge. ALT, alanine aminotransferase; AST, aspartate aminotransferase; CNS, central nervous system; HNL, histiocytic necrotizing lymphadenitis; HLH, hemophagocytic lymphohistiocytosis; LDH, lactate dehydrogenase; PICU, pediatric intensive care unit; sCD25, soluble CD25; SLE, systemic lupus erythematosus.

### 3.2 HLH-2004 criteria fulfillment

Patient-level HNL clinical features and HLH-2004 criteria fulfillment are shown in **Table 2**. All 7 patients met ≥5 of 8 criteria: 3 patients (Cases 1, 2, and 3) met 7 criteria, 2 (Cases 6 and 7) met 6, and 2 (Cases 4 and 5) met 5. Fever, ≥2-lineage cytopenia, ferritin ≥500 μg/L, and hypertriglyceridemia and/or hypofibrinogenemia were present in all patients. Hemophagocytosis on bone marrow examination was identified in 4 of 7. Functional NK-cell activity was documented as low in Case 1 and was not measured in the remaining patients. sCD25 exceeded the HLH-2004 threshold in all 5 measured patients.

**Table 2:** Patient-level HNL clinical features and fulfillment of the HLH-2004 diagnostic criteria.

Feature	Case 1	Case 2	Case 3	Case 4	Case 5	Case 6	Case 7
HNL clinical features							
Lymphadenopathy*	✓	✓	✓	✓	✓	✓	✓
Rash*	✓	×	✓	✓	✓	×	×
Hepatomegaly*	✓	✓	✓	✓	✓	✓	×
HLH-2004 diagnostic criteria							
Fever ≥38.5°C*	✓	✓	✓	✓	✓	✓	✓
Splenomegaly*	×	✓	✓	×	✓	✓	✓
Cytopenia affecting ≥2 lineages	✓	✓	✓	✓	✓	✓	✓
Hypertriglyceridemia and/or hypofibrinogenemia	✓	✓	✓	✓	✓	✓	✓
Hemophagocytosis in bone marrow or tissue	✓	✓	✓	×	×	×	✓
Low or absent NK-cell activity	✓	NT	NT	NT	NT	NT	NT
Ferritin ≥500 ng/mL	✓	✓	✓	✓	✓	✓	✓
sCD25 ≥2,400 U/mL	✓	✓	✓	✓	NT	✓	NT
Number of criteria fulfilled	7	7	7	5	5	6	6

✓feature present or criterion fulfilled; ×feature absent or criterion not fulfilled; NT, not tested. *Indicates clinical features that may occur in both HNL and HLH/MAS. HLH, hemophagocytic lymphohistiocytosis; HNL, histiocytic necrotizing lymphadenitis; MAS, macrophage activation syndrome; NK, natural killer; sCD25, soluble CD25.

### 3.3 Hematologic and biochemical findings

The median hemoglobin nadir was 64 g/L (range, 52–69), the median platelet nadir was 51 × 10^9^/L (range, 20-120), and the median absolute neutrophil count nadir was 0.64 × 10^9^/L (range, 0.05-6.06). The median peak ferritin concentration was 8,205 ng/mL (range, 1,228–16,500). The median peak alanine aminotransferase and aspartate aminotransferase levels were 361 U/L (range, 95–2,525) and 478 U/L (range, 120–7,960), respectively. The median peak fasting triglyceride level was 5.11 mmol/L (range, 2.29–5.68), the median fibrinogen nadir was 1.22 g/L (range, 0.84–2.48), and the median peak lactate dehydrogenase level was 1,851 U/L (range, 573–2,809). Among the five patients with sCD25 testing, the median concentration was 17,744 U/mL (range, 3,474–43,308); sCD25 was not measured in Cases 5 and 7. Complete patient-level values are provided in **Supplementary Table S1**.

### 3.4 Serum cytokine profile

Serum cytokines were measured in 6 of 7 patients; Case 5 was not tested. IFN-γ was above the institutional reference range in all 6 tested patients (range, 6.94–749.34 pg/mL), with the highest value in Case 3. IL-10 was elevated in 5 of 6 tested patients (range, 5.94–90.16 pg/mL), and IL-6 was elevated in all 6 tested patients (range, 49.15–1,040.05 pg/mL). TNF-α exceeded the upper institutional reference limit only in Case 1 (5.54 pg/mL; upper limit, 5.35 pg/mL); values were within the reference range in the other five tested patients. Complete patient-level cytokine values are presented in **Table 3**.

**Table 3:** Patient-level pathological, virological, autoimmune, cytokine, and lymphocyte-subset findings.

Variable	Case 1	Case 2	Case 3	Case 4	Case 5	Case 6	Case 7
Pathology and virology							
Biopsy site	Cervical	Inguinal	Inguinal	Inguinal	Cervical	Cervical	Cervical
Days from admission to biopsy	5	2	16	49	10	13	8
HNL confirmed	Y	Y	Y	Y†	Y	Y	Y
Bone marrow hemophagocytosis	Y	Y	Y	N	N	N	Y
Lymph node EBER	Negative	Scattered positive cells	Negative	Negative	Negative	Negative	Negative
Plasma EBV DNA at onset	Not detectable	Not detectable	Detectable	Not detectable	Not detectable	Not detectable	Not detectable
CMV PCR (blood)	Negative	Negative	Negative	Negative	Negative	Negative	Negative
Autoimmune markers							
ANA	Negative	Negative	Negative	Positive	Positive	Negative	Positive (1:1280)
Anti-dsDNA	Negative	Negative	Negative	Positive (1:160)	Positive	Negative	Negative
ENA profile	Negative	Negative	Negative	SSA+/CCP+	Histone+/nucleosome+/pANCA+	Negative	nRNP+/pANCA weak+
C3 (g/L; ref 0.9–1.8)	1.83	0.64	1.83	0.25	0.16	1.12	0.55
C4 (g/L; ref 0.1–0.4)	0.35	0.15	0.45	0.09	0.05	0.40	0.14
Clinical SLE diagnosis	N	N	N	Y	Y	N	Y
Timing of SLE diagnosis	NA	NA	NA	Approximately 92 days after admission	Day 11 after admission	NA	Day 6 after admission
Full serum cytokine panel							
IFN-γ (pg/mL; ref 0–6.56)	14.84	6.94	749.34	12.85	NA	134.90	20.72
IL-10 (pg/mL; ref 0–8.14)	8.98	40.73	54.70	61.53	NA	90.16	5.94
IL-6 (pg/mL; ref 0–8.88)	49.15	111.70	112.21	1,040.05	NA	62.06	320.93
TNF-α (pg/mL; ref 0–5.35)	5.54	1.18	1.60	2.33	NA	3.40	1.61
IL-4 (pg/mL; ref 0–4.62)	4.28	0.33	1.31	2.14	NA	3.67	0.68
Lymphocyte subsets							
Total lymphocytes (cells/μL)	1,153	450	1,164	252	310	8,026	605
NK proportion (%; ref 8–34)	9.83	9.21	3.78	1.17	6.82	3.85	1.11
NK absolute count (cells/μL)	113	41.4	44.0	2.95	21.1	309	6.71
CD4 proportion (%)	45.5	25.1	22.5	32.0	26.9	17.8	57.0
CD8 proportion (%)	36.1	53.3	66.0	9.57	44.0	74.8	29.6
NK proportion below reference range	N	N	Y	Y	Y	Y	Y
CD8 proportion >60%	N	N	Y	N	N	Y	N

†HNL was confirmed after repeated institutional pathological review in Case 4. Peripheral-blood NK-cell proportion reflects flow-cytometric enumeration and is not equivalent to functional NK-cell activity; it was not used to fulfill the HLH-2004 NK-cell activity criterion. ANA, antinuclear antibody; anti-dsDNA, anti-double-stranded DNA; CCP, cyclic citrullinated peptide; CMV, cytomegalovirus; EBER, Epstein-Barr virus-encoded small RNA *in-situ* hybridization; EBV, Epstein-Barr virus; ENA, extractable nuclear antigen; HNL, histiocytic necrotizing lymphadenitis; IFN-γ, interferon-gamma; IL, interleukin; NA, not available/not applicable; NK, natural killer; nRNP, nuclear ribonucleoprotein; pANCA, perinuclear antineutrophil cytoplasmic antibody; ref, institutional reference range; SLE, systemic lupus erythematosus; TNF-α, tumor necrosis factor-alpha; Y/N, yes/no.

### 3.5 Immunological findings

Autoimmune markers and complete lymphocyte-subset findings are presented in **Table 3**. At the index admission, antinuclear antibody (ANA) was positive in 3 of 7 patients (Cases 4, 5, and 7), with the highest titer in Case 7 (1:1280). Anti-double-stranded DNA antibody was positive in Cases 4 and 5. C3 was below the institutional reference range in 4 patients (Cases 2, 4, 5, and 7), and C4 was below the reference range in 2 (Cases 4 and 5). Extractable nuclear antigen profiles differed among the three ANA-positive patients. Detailed immunoglobulin and autoantibody profiles are provided in **Supplementary Table S5**.

Admission NK-cell proportions ranged from 1.11% to 9.83%, and Cases 3–7 were below the local laboratory reference range (8%–34%). CD8-positive T-cell proportions exceeded 60% in Cases 3 and 6 (66.0% and 74.8%, respectively). Case 6 had the highest absolute lymphocyte count (8,026 cells/μL), with CD8-positive T cells accounting for 74.8% of lymphocytes.

### 3.6 Lymph node pathology

Lymph node biopsy was performed 2–49 days after admission; four biopsies were obtained from cervical nodes and three from inguinal nodes (**Table 3**). HNL was documented in the final institutional pathology report for all seven patients. Where immunohistochemistry was performed, CD68- and CD163-positive histiocytes were identified within karyorrhectic foci, and the available B-cell and T-cell markers did not support lymphoma. The final lymph node assessment in Case 1 supported HNL and described fragmented lymph node tissue with histiocytic proliferation and diffuse CD68 positivity. Bone marrow examination demonstrated histiocytes engulfing erythrocytes, fulfilling the hemophagocytosis criterion of HLH-2004. Per-patient histology and immunohistochemistry results are provided in **Supplementary Table S2**. Plasma EBV DNA was detectable in one of seven patients, whereas blood CMV PCR was negative in all seven patients. Lymph node EBER was negative in six patients and showed scattered positive cells in one.

### 3.7 Treatment, complications, and acute outcomes

Treatment regimens, PICU duration, and acute outcomes are summarized in **Table 4**. All seven patients received intravenous systemic glucocorticoids and intravenous immunoglobulin; none received etoposide. Additional immunosuppressive or immunomodulatory therapies were used in selected patients, including cyclosporine A in Cases 4 and 6, tocilizumab in Case 4, and mycophenolate mofetil, hydroxychloroquine, and belimumab in Case 7 with concurrent SLE. Four patients (Cases 1, 2, 4, and 7) required PICU admission for 9–55 days. Supportive therapies included plasma exchange in Cases 1, 4, and 7, mechanical ventilation in Cases 1 and 2, and vasoactive drug support in Case 7. Additional recorded complications included a small coronary aneurysm in Case 3 and intestinal necrosis with encephalopathy in Case 4. At discharge, complete remission of HLH was documented in six patients, whereas partial remission was documented in Case 2; no in-hospital deaths occurred.

**Table 4:** Treatment, acute outcomes, and follow-up outcomes.

Variable	Case 1	Case 2	Case 3	Case 4	Case 5	Case 6	Case 7
Treatment							
Systemic glucocorticoids	Y	Y	Y	Y	Y	Y	Y
Peak glucocorticoid record*	MP 4 mg/kg	MP 2 mg/kg	Dex 2.5 mg total	Dex 10 mg	MP 5 mg/kg	MP 5 mg/kg	MP 10 mg/kg
IVIG cumulative dose (g/kg)	2	2	4	>2	1	2	2
Cyclosporine A	N	N	N	Y	N	Y	N
Tocilizumab (anti-IL-6R)	N	N	N	Y	N	N	N
Mycophenolate mofetil	N	N	N	N	N	N	Y
Hydroxychloroquine	N	N	N	N	N	N	Y
Belimumab	N	N	N	N	N	N	Y
Etoposide	N	N	N	N	N	N	N
Organ support and acute complications							
PICU admission	Y	Y	N	Y	N	N	Y
PICU duration (days)	9	20	NA	55	NA	NA	19
Plasma exchange	Y	N	N	Y	N	N	Y
Number of plasma-exchange sessions	2	0	0	NR	0	0	NR
Mechanical ventilation	Y	Y	N	N	N	N	N
Vasoactive drug support	N	N	N	N	N	N	Y
Other major complications	None recorded	None recorded	Small coronary aneurysm	Intestinal necrosis/encephalopathy	None recorded	None recorded	None recorded
Outcomes and follow-up							
Discharge outcome	CR	PR	CR	CR	CR	CR	CR
In-hospital death	N	N	N	N	N	N	N
Post-discharge follow-up available	Y	N	Y	Y	Y	Y	Y
Follow-up duration (months)	28	NA	12	19	73	7	38
HLH recurrence	N	Not assessable	N	N	N	N	N
Clinical SLE diagnosis	N	No before loss to follow-up	N	Y	Y	N	Y
Days from admission to SLE diagnosis	NA	NA	NA	~92	11	NA	6
Status at last follow-up	Alive; no documented HLH recurrence or SLE	Lost to follow-up after discharge	Alive; no documented HLH recurrence or SLE	Alive with SLE	Alive with SLE	Alive; no active disease documented	Alive with SLE

*Peak glucocorticoid regimen is shown as recorded in source medical records and was not converted across agents. Acute remission status was recorded according to the treating team’s assessment at discharge. CR, complete remission; Dex, dexamethasone; HLH, hemophagocytic lymphohistiocytosis; IVIG, intravenous immunoglobulin; MP, methylprednisolone; NA, not applicable or unavailable; NR, number not recorded in the available source records; PICU, pediatric intensive care unit; PR, partial remission; SLE, systemic lupus erythematosus.

### 3.8 SLE diagnosis and follow-up outcomes

Post-discharge follow-up was available for six patients; Case 2 was lost to follow-up. The median follow-up duration was 23.5 months (range, 7–73 months). No HLH recurrence was documented during the available follow-up. Clinical SLE was diagnosed in three patients during the index hospitalization: Cases 7 and 5 at 6 and 11 days after admission, respectively, and Case 4 approximately 92 days after admission. Detailed clinical and serological features of these three patients are provided in **Supplementary Table S4**.

## 4 Discussion

This single-center retrospective case series describes seven children with biopsy-confirmed HNL who fulfilled HLH-2004 criteria. The principal findings were marked acute hyperinflammation, PICU admission in four patients, and clinical SLE diagnoses in three patients during the index hospitalization. No in-hospital deaths occurred, and no HLH recurrence was documented among the six patients with follow-up. Admission NK-cell proportions were below the local laboratory reference range in five of seven patients.

Previous studies indicate that HLH is an uncommon but clinically important complication of HNL. A pediatric series of 13 patients reported heterogeneous presentations and generally favorable outcomes, although one child died and two survivors developed autoimmune disease (12). Macrophage activation syndrome has also been reported in children with HNL (13). In the adult five-patient HNL-HLH series by Chen and colleagues, two patients were diagnosed with SLE during follow-up (14). Consistent with these reports, our series included only patients with biopsy-confirmed HNL and provides detailed patient-level descriptions of HLH-2004 criteria, treatment, acute outcomes, and SLE diagnoses. A descriptive comparison of the present cohort with these pediatric and adult series is provided in **Supplementary Table S3**.

A clinically important finding was that all three SLE diagnoses were made during the index hospitalization, at 6, 11, and approximately 92 days after admission. Thus, autoimmune features were recognized either early or later during the same clinical episode. Published HNL-SLE case series, systematic reviews, pediatric HNL-SLE-MAS reports, and adult HNL-HLH follow-up data also support clinical overlap between these entities (5–7, 14). Mechanistically, plasmacytoid dendritic cells, cytotoxic T-cell responses, and type I interferon-related features described in HNL may provide a plausible immunological context for HNL-SLE overlap (19, 20). Children with HNL-HLH and ANA or anti-dsDNA positivity, hypocomplementemia, or relevant mucocutaneous, renal, or neurological manifestations may warrant rheumatologic assessment and longitudinal follow-up. When HLH/MAS is suspected or inflammation progresses, the initial evaluation should include the hyperinflammatory state, potential triggers, organ dysfunction, and need for multidisciplinary input (21).

Low NK-cell proportions were observed in most patients in this cohort. Previous pediatric HLH studies have also reported peripheral lymphocyte-subset abnormalities, including decreased NK-cell proportions, increased CD8-positive T-cell proportions, and decreased CD4/CD8 ratios (22, 23). Peripheral-blood NK-cell proportion is not equivalent to functional NK-cell activity. Because NK-cell function was assessed in only one patient, larger studies are needed to determine its relationship with disease severity and subsequent autoimmune outcomes.

Lymph node pathology was central both for confirming HNL and for excluding important differential diagnoses in a hyperinflammatory setting. HNL-HLH may overlap clinically with infectious lymphadenitis, lymphoma, SLE lymphadenitis, and other necrotizing lymphadenitides; persistent fever, hepatosplenomegaly, lymphadenopathy, cytopenias, and hyperferritinemia alone are insufficient to separate these conditions. All seven patients underwent lymph-node biopsy, and the final institutional pathology assessments incorporated the available histological and immunohistochemical findings. Biopsy specimens were obtained from cervical or inguinal nodes. HNL was confirmed in Case 4 after repeated pathological review. In Case 3, scattered weak cytoplasmic ALK staining did not support a diagnosis of ALK-positive lymphoma in the final pathology assessment. These cases underscore the value of adequate tissue sampling, immunohistochemistry, and integrated hematopathological interpretation.

Treatment and supportive-care data indicate substantial acute disease burden despite the absence of in-hospital mortality. Four patients required PICU care, three underwent plasma exchange, two required mechanical ventilation, and one required vasoactive support. All patients received intravenous glucocorticoids and IVIG. Selected patients also received cyclosporine A, tocilizumab, or SLE-directed immunotherapy; none received etoposide. Initial empiric immunomodulatory therapy for rapidly progressive HLH/MAS may include the IL-1 inhibitor anakinra, high-dose glucocorticoids, and/or IVIG (21). However, anakinra was not used in this cohort, and our data cannot determine its role in HNL-HLH. Because treatment was nonstandardized and the series lacked a comparator group, these data do not support comparisons among treatment strategies or establish an etoposide-sparing approach. Patients with suspected familial or genetic HLH, or with severe, persistent, relapsed, or refractory disease, may require HLH-directed regimens, including etoposide-containing therapy. Belimumab in Case 7 was recorded in the context of concurrent SLE; this observation should not be interpreted as evidence for routine belimumab use in HNL-HLH (24).

This study has several limitations. First, this retrospective single-center study included only seven patients and lacked a comparator group; referral patterns may also have enriched the cohort for severe or diagnostically complex cases. Second, treatment was not standardized, precluding comparison of corticosteroid intensity, IVIG use, plasma exchange, biologic therapy, or etoposide-free management. Third, several variables were unavailable or were not assessed uniformly, including cytokines, sCD25, functional NK-cell activity, immunohistochemistry panels, and longitudinal rheumatologic outcomes. Type I interferon signature testing was not performed. Fourth, genetic testing for HLH-associated variants was not performed, precluding assessment of an underlying genetic predisposition and whether HNL may have served as a trigger for HLH in genetically susceptible patients. Genetic testing should therefore be considered during follow-up to support genetic counseling for patients and their families. Future multicenter pediatric studies should standardize HLH-2004 documentation, cytotoxicity and genetic testing, treatment and organ-support variables, and long-term rheumatologic follow-up.

## 5 Conclusion

In this seven-patient pediatric series, biopsy-confirmed HNL co-occurred with HLH-2004-defined hyperinflammation, and four patients required intensive care. Three patients were clinically diagnosed with SLE during the index hospitalization, while no in-hospital deaths were recorded. These observations highlight the importance of pathological confirmation, assessment of hyperinflammatory severity, and rheumatologic follow-up in children with HNL-HLH. Larger multicenter studies are needed to determine the prognostic relevance of immune-profile abnormalities and to inform treatment-intensity stratification.

## Data Availability

The original contributions presented in the study are included in the article/**Supplementary Material**. Further inquiries can be directed to the corresponding authors.
