# Supplementary material for: Kikuchi-Fujimoto disease with hemophagocytic lymphohistiocytosis in children: a seven-patient case series
Source: Front Immunol. 2026 Sep 30;17:1935403. doi: 10.3389/fimmu.2026.1935403 (PMC13661800; doi:10.3389/fimmu.2026.1935403)
Supplement: Supplementary file 1 [file SupplementaryFile1.docx]

**Supplementary Material**

**Supplementary Table S1. Full laboratory characteristics during the index hospitalization (peak or nadir values as applicable).**

| **Variable** | **Case 1** | **Case 2** | **Case 3** | **Case 4** | **Case 5** | **Case 6** | **Case 7** |
| --- | --- | --- | --- | --- | --- | --- | --- |
| **WBC nadir (×10⁹/L)** | 10.7 | 3.2 | 1.9 | 1.8 | 0.8 | 2.7 | 1.0 |
| **ANC nadir (×10⁹/L)** | 6.06 | 2.27 | 0.05 | 0.64 | 0.29 | 0.12 | 0.64 |
| **Lymphocyte nadir (×10⁹/L)** | 1.22 | 0.70 | 1.65 | 0.26 | 0.28 | 1.15 | 0.31 |
| **Hb nadir (g/L)** | 69 | 64 | 64 | 52 | 65 | 63 | 66 |
| **PLT nadir (×10⁹/L)** | 98 | 35 | 51 | 20 | 58 | 120 | 50 |
| **CRP peak (mg/L)** | 87.32 | 143.59 | 10.16 | 144.89 | 1.80 | 84.74 | 89.06 |
| **ESR peak (mm/h)** | 56 | 86 | 88 | 94 | 17 | 18 | 36 |
| **ALT peak (U/L)** | 107 | 174 | 963 | 2,032 | 95 | 2,525 | 361 |
| **AST peak (U/L)** | 290 | 239 | 1,073 | 7,960 | 120 | 2,176 | 478 |
| **LDH peak (U/L)** | 2,057 | 1,851 | 1,214 | 1,730 | 573 | 2,809 | 2,126 |
| **Albumin nadir (g/L)** | 28.1 | 26.4 | 28.8 | 20.3 | 19.9 | 26.3 | 23.3 |
| **Total bilirubin peak (μmol/L)** | 14.3 | 53.2 | 29.8 | 332.8 | 4.1 | 90.0 | 18.8 |
| **Fibrinogen nadir (g/L)** | 1.56 | 0.84 | 2.48 | 0.88 | 1.71 | 1.16 | 1.22 |
| **Triglyceride peak (mmol/L)** | 5.11 | 2.29 | 5.52 | 3.56 | 5.11 | 3.17 | 5.68 |
| **Ferritin peak (ng/mL)** | 14,468 | 6,783 | 16,500 | 1,992 | 1,228 | 16,500 | 8,205 |
| **D-dimer peak (μg/mL)** | 10.59 | 16.50 | NA | 16.88 | 1.49 | NA | 10.40 |
| **sCD25 (U/mL; HLH-2004 threshold ≥2,400)** | 14,103 | 17,744 | 33,410 | 43,308 | NA | 3,474 | NA |

Note: Values are peak or nadir values during the index hospitalization, as clinically appropriate. Reference intervals are not displayed because they vary by age and assay; values are presented descriptively. sCD25 is reported in U/mL; Cases 5 and 7 were not tested. ALT, alanine aminotransferase; ANC, absolute neutrophil count; AST, aspartate aminotransferase; CRP, C-reactive protein; ESR, erythrocyte sedimentation rate; Hb, hemoglobin; LDH, lactate dehydrogenase; NA, not available; PLT, platelet; sCD25, soluble CD25; WBC, white blood cell count.

**Supplementary Table S2. Detailed lymph node histology and immunohistochemistry per patient.**

| **Case** | **Histology and IHC summary from the final institutional pathology assessment** |
| --- | --- |
| **Case 1** | CD3/PAX5 show normal B/T-zone distribution; CD21 demonstrates largely preserved follicular structures; BCL2 and BCL6 are unremarkable; Ki-67 approximately 30% in follicles and 35% interfollicular. CD68 diffusely positive; MUM1 occasional positive; MPO scattered positive; S100 occasional positive. CD30, TdT, desmin, myogenin, ALK, CD56, EBER, CD117, CD34, CD1a negative. PAS and acid-fast negative. |
| **Case 2** | EBER shows scattered positive cells. CD21 highlights disrupted follicular dendritic cell network with residual positive cells. CD3 and CD20 positive in expected distributions. BCL2 partially positive; CD10 largely negative; BCL6 with small residual positive cells; Ki-67 approximately 40%. ALK negative; CD30 scattered weak positive; CD163 positive in histiocytes. |
| **Case 3** | CD68 and CD163 positive in histiocytic infiltrate. CD3/CD5 highlight paracortical T cells; CD4 and CD8 partially positive. PAX5/CD20 positive in residual follicular zones. CD30 negative; ALK weak cytoplasmic positivity in scattered cells. CD21 focally residual. CD10 occasional positive; TIA1 partially positive; BCL6 with few residual positive cells. Ki-67 up to approximately 80%. EBER negative. |
| **Case 4** | CD20 and PAX5 occasional positive; CD3 and CD7 positive in paracortical T cells; CD68 and S100 positive in histiocytes. Ki-67 approximately 15%. CD21 with residual follicular dendritic cell network. CD30 positive in scattered cells. ALK negative; EBER negative. The diagnosis of HNL was confirmed after repeated institutional pathological review. |
| **Case 5** | PAS and Gomori methenamine silver stains negative. CD68 and CD163 positive in histiocytes. CD20 highlights follicular B cells; CD3 paracortical T cells; CD21 follicular dendritic cell network preserved; Ki-67 high in follicles. ALK negative; CD30 focal scattered weak positive. EBER negative. |
| **Case 6** | CD20, CD3, BCL2, and CD21 showed unremarkable distributions. CD68 and CD163 were positive, and S100 showed scattered positivity. Ki-67 was approximately 80% in follicles, 15% in interfollicular areas, and 40% in disrupted areas. CD30 showed occasional positivity in scattered cells; ALK was negative; and CD1a, cyclin D1, and langerin showed occasional positivity in scattered cells. |
| **Case 7** | EBER was negative. Although a complete immunohistochemistry panel was not performed, the final institutional histopathological assessment supported a diagnosis of HNL. |

Note: ALK, anaplastic lymphoma kinase; BCL, B-cell lymphoma; CD, cluster of differentiation; EBER, Epstein-Barr virus-encoded small RNA in-situ hybridization; HNL, histiocytic necrotizing lymphadenitis; IHC, immunohistochemistry; MPO, myeloperoxidase; MUM1, multiple myeloma oncogene 1; PAS, periodic acid-Schiff stain; PAX5, paired box protein 5; TdT, terminal deoxynucleotidyl transferase; TIA1, T-cell intracellular antigen 1.

**Supplementary Table S3. Descriptive comparison of the present case series with selected published HNL-HLH cohorts.**

| **Variable** | **Present cohort** | **Yang et al., 2021** | **Chen et al., 2024** |
| --- | --- | --- | --- |
| **Study design and setting** | Single-center retrospective pediatric case series | Single-center retrospective pediatric case-control study | Single-center retrospective adult case series |
| **Country** | China | China | China |
| **Recruitment period** | January 2020–December 2025 | January 2007–October 2019 | October 2010–June 2015 |
| **Number of HNL-HLH patients** | 7 | 13 | 5 |
| **Age** | Median 7 years (range, 1–13) | Mean 8.1 ± 1.2 years | Median 37 years |
| **Sex, male:female** | 4:3 | 9:4 | 2:3 |
| **Case definition** | Biopsy-confirmed HNL and ≥5 of 8 HLH-2004 criteria | Biopsy-confirmed HNL and HLH-2004-defined HLH; other causes excluded | Histopathologically confirmed HNL and HLH-2004-defined HLH; other causes excluded |
| **Treatment overview** | Intravenous glucocorticoids and IVIG in 7/7; etoposide in 0/7 | Additional immunosuppressive drugs or chemotherapy in 3/13 because of poor HLH control | Glucocorticoid-containing regimens in 5/5; IVIG in 1/5 and cyclophosphamide in 1/5 |
| **Mortality** | 0/7 | 1/13 | 0/5 |
| **Autoimmune outcome** | SLE in 3/7; all diagnoses occurred during the index hospitalization, at 6, 11, and approximately 92 days after admission | Autoimmune disease in 2/12 surviving patients | SLE in 2/5 during follow-up |

Note: Comparison is descriptive only; no inferential comparison was performed. Definitions, treatment reporting, and follow-up differed across studies. Sources: Yang Y, Lian H, Ma H, et al. J Pediatr. 2021;229:267–274.e3. doi:10.1016/j.jpeds.2020.08.063; Chen Q, Zhang J, Huang H, et al. Immun Inflamm Dis. 2024;12:e1202. doi:10.1002/iid3.1202. HLH, hemophagocytic lymphohistiocytosis; HNL, histiocytic necrotizing lymphadenitis; IVIG, intravenous immunoglobulin; SLE, systemic lupus erythematosus.

**Supplementary Table S4. Clinical and serological features of the three patients clinically diagnosed with SLE.**

| **Feature** | **Case 4** | **Case 5** | **Case 7** |
| --- | --- | --- | --- |
| Clinical SLE diagnosis recorded in source records | Yes | Yes | Yes |
| Relative timing from index admission | Approximately 92 days after admission | Day 11 after admission | Day 6 after admission |
| ANA | Positive | Positive | Positive (1:1280) |
| Anti-dsDNA | 1:160 | Positive | Negative |
| Complement profile | Low C3 and low C4 | Low C3 and low C4 | Low C3 |
| ENA / other autoantibody pattern | SSA+; CCP+ | Anti-histone+; anti-nucleosome+; pANCA+ | Anti-nRNP+; weak pANCA+ |
| Clinical features shared by HLH and SLE | Fever, cytopenias, encephalopathy | Fever and cytopenias | Fever and cytopenias |

Note: Features are summarized from extracted source records. ANA, antinuclear antibody; anti-dsDNA, anti-double-stranded DNA; CCP, cyclic citrullinated peptide; ENA, extractable nuclear antigen; HLH, hemophagocytic lymphohistiocytosis; nRNP, nuclear ribonucleoprotein; pANCA, perinuclear antineutrophil cytoplasmic antibody; SLE, systemic lupus erythematosus; SSA, Sjögren syndrome-related antigen A.

**Supplementary Table S5. Autoimmune serology and immunoglobulin profile at the index admission.**

| **Variable** | **Case 1** | **Case 2** | **Case 3** | **Case 4** | **Case 5** | **Case 6** | **Case 7** |
| --- | --- | --- | --- | --- | --- | --- | --- |
| **ANA** | Neg | Neg | Neg | Pos | Pos | Neg | Pos 1:1280 |
| **Anti-dsDNA** | Neg | Neg | Neg | 1:160 | Pos | Neg | Neg |
| **ENA profile** | Neg | Neg | Neg | SSA+; CCP+ | Anti-histone+; anti-nucleosome+; pANCA+ | Neg | Anti-nRNP+; weak pANCA+ |
| **C3 (g/L; ref 0.9-1.8)** | 1.83 | 0.64 | 1.83 | 0.25 | 0.16 | 1.12 | 0.55 |
| **C4 (g/L; ref 0.1-0.4)** | 0.35 | 0.15 | 0.45 | 0.09 | 0.05 | 0.40 | 0.14 |
| **IgE (IU/mL)** | 682 | 2,540 | 377 | 673 | 54 | 589 | 992 |
| **IgG (g/L)** | 9.36 | 10.60 | 18.60 | 20.70 | 21.70 | 15.90 | 23.70 |
| **IgA (g/L)** | 2.57 | 1.48 | 1.00 | 2.73 | 1.95 | 2.31 | 5.09 |
| **IgM (g/L)** | 1.97 | 0.88 | 1.10 | 2.20 | 1.00 | 2.18 | 1.28 |
| **Clinical autoimmune diagnosis** | No | No | No | SLE | SLE | No | SLE |

Note: Immunoglobulin values are presented descriptively because age-specific institutional reference intervals vary and are not displayed in this table. ANA, antinuclear antibody; anti-dsDNA, anti-double-stranded DNA; C3, complement component 3; C4, complement component 4; CCP, cyclic citrullinated peptide; ENA, extractable nuclear antigen; Ig, immunoglobulin; nRNP, nuclear ribonucleoprotein; pANCA, perinuclear antineutrophil cytoplasmic antibody; SLE, systemic lupus erythematosus; SSA, Sjögren syndrome-related antigen A.
